# The Therapeutic Effects of *Dendropanax morbiferus Lév*. Water Leaf Extracts in a Rheumatoid Arthritis Animal Model

**DOI:** 10.3390/antiox14050548

**Published:** 2025-05-01

**Authors:** Dongho Lee, Min Jung Kim, Chang-Soo Cho, Ye Jin Yang, Jin-Kyung Kim, Ryounghoon Jeon, Sang-Hyun An, Kwang Il Park, Kwangrae Cho

**Affiliations:** 1 Department of Anesthesiology and Pain Medicine, College of Medicine, Inje University, Gimhae 47392, Republic of Korea; akadinger.dl@gmail.com; 2 College of Veterinary Medicine, Gyeongsang National University, Jinju 52828, Republic of Korea; min-jung0102@gnu.ac.kr (M.J.K.); feeljcs1917@gmail.com (C.-S.C.); yang93810@gnu.ac.kr (Y.J.Y.); 3 Department of Dental Hygiene, Daegu Health College, Daegu 41453, Republic of Korea; jkk0621@gmail.com; 4 Preclinical Research Center, Daegu-Gyeongbuk Medical Innovation Foundation (K-MEDI hub), Daegu 41061, Republic of Korea; rhjeon@kmedihub.re.kr (R.J.); ash4235@kmedihub.re.kr (S.-H.A.)

**Keywords:** rheumatoid arthritis, *Dendropanax morbiferus Lév*. leaf, CHON-001 cells, Balb/c mouse, micro-computed tomography, anti-inflammatory

## Abstract

(1) Background: Rheumatoid arthritis (RA) is a chronic inflammatory condition known for its symptoms of joint damage and cartilage breakdown. Current treatments frequently result in adverse effects and show restricted efficacy in the long term. *Dendropanax morbiferus*, a plant recognized for its bioactive properties, demonstrates promise in the treatment of inflammatory conditions. The objective of this study was to examine the therapeutic properties of *Dendropanax morbiferus Lév.* water extract (DMWE) in RA through the utilization of in vitro and in vivo models. (2) Methods: Ultra-high-performance liquid chromatography (UPLC) analysis was used to identify bioactive compounds in DMWE. Antioxidant activity was evaluated using 1,1-diphenyl-2-picrylhydrazyl (DPPH) and 2,2-azino-bis(3-ethylbenzothiazoline-6-sulfonic acid) diammonium salt (ABTS) radical-scavenging assays. The in vitro experiments involved the treatment of CHON-001 cells with DMWE in order to assess its impacts on inflammation and matrix metalloproteinase (MMP) expression. The impact of DMWE on the Janus Kinase 2 (JAK2) and Signal Transducer and Activator of Transcription (STAT) signaling pathways was also assessed. RA was induced in Balb/c mice who were subsequently treated with varying doses of DMWE to assess its impact on joint morphology, edema, and body weight. (3) Results: DMWE demonstrated substantial antioxidant activity and hindered the expression of MMP-2 and MMP-8 in chondrocytes treated with IL-1β. It additionally inhibited the JAK2/STAT pathway and diminished inflammatory responses. Treatment with DMWE in living organisms led to a decrease in joint swelling, improved weight regains, and maintained joint structure, with higher doses exhibiting effects similar to those of the positive control, dexamethasone (Dexa). (4) Conclusions: DMWE was found to have excellent in vitro antioxidant and anti-inflammatory activities. In an RA-induced mouse model, DMWE-3 (500 mg/kg BW) was found to effectively treat RA by reducing the concentration of pro-inflammatory factors and preventing joint deformation.

## 1. Introduction

Rheumatoid arthritis (RA) is a persistent autoimmune condition that predominantly affects the joints, but may also have an impact on other tissues and organs [1]. Chronic inflammation is characterized by the infiltration of immune cells, including macrophages, T cells, and B cells, which release pro-inflammatory mediators and enzymes that degrade extracellular matrix components [2]. This process harms the cartilage and bones within the joints, resulting in joint destruction and subsequent functional impairment and deformation. RA is mostly found in individuals between the ages of 30 and 60, with a higher incidence among women than men. If left without proper medical intervention, this condition may advance, ultimately resulting in joint deformities and complications affecting various systems of the body.

An important factor in the development of RA lies in the communication between immune system cells such as macrophages and T cells with cells in the affected joint [3]. Inflammatory cytokines such as tumor necrosis factor-α (TNF-α), interleukin-1β (IL-1β), and interleukin-6 (IL-6) play a crucial role in the onset and advancement of RA. Recent studies indicate that antigen-activated CD4^+^ T cells, monocytes, macrophages, and synovial fibroblasts produce cytokines, which subsequently trigger the release of matrix metalloproteinases (MMPs) by chondrocytes, fibroblasts, and osteoclasts [4,5]. Subsequently, the gradual destruction of the joints and impairment of function occur due to the erosion of bone and cartilage.

The exact cause of RA is currently unknown, but it is thought to result from a combination of genetic susceptibility, environmental factors such as smoking and infection, and hormonal influences. The main goals of treatment include disease-modifying antirheumatic drugs and biological therapies that target particular immune pathways. In recent years, preclinical trials have shown that natural plant extracts and compounds can potentially reduce the symptoms associated with RA [6,7].

*Dendropanax morbiferus* has been reported to possess various health benefits, including antioxidant [8,9], antimicrobial [10], antidiabetic [11], and hepatoprotective properties, as well as effects related to metabolic disorders and pharmacological impacts [12]. However, despite these promising advantages, research on the potential therapeutic efficacy of *Dendropanax morbiferus* leaf water extract (DMWE) in the treatment of RA has been lacking.

With the critical involvement of chondrocytes in the pathogenesis of RA [13,14], the aim of this research is to assess the therapeutic efficacy and mechanisms of action of DMWE in RA through the use of chondrocyte cultures and animal model studies. We aim to investigate the potential of DMWE as a novel therapeutic agent for RA by examining its ability to modulate inflammatory Janus Kinase 2 (JAK2)/Signal Transducer and Activator of Transcription (STAT) pathways and regulate the expression of enzymes such as MMPs.

The main components of a hot water extract from DMWE were identified using ultra-high-performance liquid chromatography–time-of-flight/tandem mass spectrometry (UPLC-TOF/MS-MS), and its antioxidant and anti-inflammatory properties were verified using in vitro studies. In addition, mice were orally given DMWE for a total of five weeks, with administration commencing two weeks before and continuing for three weeks after the initiation of RA. Macroscopic assessments were conducted to evaluate alterations induced by RA, while micro-computed tomography (micro-CT) was utilized to assess the effect on joint deformation. At the conclusion of the experiment, all animals were euthanized, and serum was separated from blood collected via cardiac puncture. Changes in the concentrations of inflammation-related cytokines (TNF-α, IL-6) in the serum were then investigated. Ultimately, the therapeutic efficacy of the DMWE for RA was confirmed based on a comprehensive analysis of macroscopic findings, micro-CT results, and changes in the levels of inflammation-related cytokines.

## 2. Materials and Methods

### 2.1. Sample Preparation

*Dendropanax morbiferus* leaves were provided by Hurim Hwangchil Co., Ltd. (Jinju, Republic of Korea), cultivated and harvested in Hadong-gun (Gyeongsangnam-do, Republic of Korea). *Dendropanax morbiferus* leaves were extracted using hot water at 95–100 °C for 4 h, and the sample was filtrated using a 100 µm micro-filter. Next, the filtered extracts were concentrated at 14–16 brix at 45 °C using an evaporator under vacuum. After being lyophilized at −40 °C for 96 h, the sample was ground into powder. The powder produced through this process was named DMWE (yield: 22.57%) (Figure S1).

### 2.2. Condition of UPLC-QTOF/MS Analysis

The DMWE was analyzed using a Nexera XS ultra-performance liquid chromatography (UPLC) system (Shimadzu; Kyoto, Japan) coupled to an X500R quadrupole time-of-flight (QTOF)/mass spectrometry (MS) system (SCIEX ExionLC AD system; Framingham, MA, USA) with an electrospray ionization (ESI) source [15]. A Pronto SIL (150 × 4.6 nm, 5 µm, 120-5 C18 SH) (Bischoff Chromatography; Leonberg, Germany) was used for UPLC with a column oven temperature of 35 °C. The flow rate was 0.5 mL/min, and the injection volume was 10 μL. The mobile phase was 0.1% formic acid in H_2_O (solution A) and 0.1% formic acid in acetonitrile (solution B). The gradient elution was as follows: from 0 to 10% solution B for 0–10 min; from 10 to 15% solution B for 10–20 min; from 15 to 20% solution B for 20–30 min; from 20 to 25% solution B for 30–40 min; from 25 to 40% solution B for 40–50 min; from 40 to 70% solution B for 50–60 min; from 5 to 95% solution B for 60~65 min. The identification of compounds using QTOF/MS was performed in both positive (ESI, 5500 V) ion modes. The conditions of MS were as follows: ion source gas 1 and gas 2, 50 and 60 psi; curtain gas, 30 psi; ion source temperature, 550 °C. The mass spectra were recorded in the mass-to-charge (*m*/*z*) range of 100–1500 for QTOF/MS and 50–1500 for MS/MS with collision energy (CE 45 V, CE spread 15 V). Data acquisition and compound identification were performed using SCIEX OS software version 3.0.0.3339.

### 2.3. Antioxidant Evaluation

The radical scavenging activities of 1,1-diphenyl-2-picrylhydrazyl (DPPH) and 2,2-azino-bis(3-ethylbenzothiazoline-6-sulfonic acid) diammonium salt (ABTS) were measured according to previously reported methods with slight modifications. For DPPH, the reagent (0.15 mM in methanol; Sigma-Aldrich, St. Louis, MO, USA) was mixed with DMWE solutions (1, 5, 10, and 50 mg/mL in distilled water) at a ratio of 2.5 mL to 1 mL, vortexed, and incubated in the dark for 30 min [16]. Absorbance was measured at 518 nm using a spectrophotometer (SpectraMax M2e, Molecular Devices, LLC, San Jose, CA, USA). For ABTS, a solution was prepared by reacting 7 mM ABTS with 2.4 mM potassium persulfate (1:1, *v*/*v*) in the dark for 16 h and diluted to an absorbance of 1.0 at 734 nm. DMWE solutions (1, 5, 10, and 50 mg/mL) were mixed with 190 µL of ABTS solution in a 96-well microplate and incubated at room temperature for 10 min, and absorbance was measured at 734 nm [17]. For both assays, distilled water and 1.0 mg/mL vitamin C served as negative and positive controls, respectively. The radical scavenging activity was calculated as a percentage using the formula.

$$\mathrm{Radical}\;\mathrm{scavenging}\;\mathrm{activity}(\%)=(\frac{{AB}_{NC}-{AB}_{sam}}{{AB}_{NC}})\times 100$$

where: AB_NC_, absorbance of negative control; AB_sam_, absorbance of sample.

### 2.4. Ln Silico Molecular Docking

The molecular docking of utin within the MMP-8 region was assessed using the 3D crystallographic structure of MMP-8 obtained from the Protein Data Bank (PDB ID: 1BZS) website. The enzyme’s crystal structure was modified using Discovery Studio Visualizer software version 17.2.0.16349, 2016, by eliminating ligands and water molecules linked to the PDB file. The docking procedure was conducted utilizing AutoDock 4.2 software with the Lamarckian Genetic Algorithm, under the assumption of rigid ligands within the macromolecule and full flexibility for the ligand. The universal gas constant in AutoDock is denoted as R, with a value of 1.987 cal/(mol·K), and T represents the temperature in Kelvin, with a default value of 298.15 K [18,19]. The software utilized for obtaining two-dimensional and three-dimensional images of the selected most stable conformation was Discovery Studio Visualizer version 21.1.0.20298.

### 2.5. Cell Culture and Cytotoxicity

The American Type Culture Collection (ATCC, University Boulevard Manassas, Manassas, VA, USA) provided the human chondrocyte CHON-001 cells, which were grown in full DMEM with 10% heat-inactivated fetal bovine serum, 100 U/mL penicillin, and 100 g/mL streptomycin. The cells were incubated at a temperature of 37 °C in a humid atmosphere with 5% CO_2_. To set the test substance treatment concentration, cultured cells were plated at a density of 1 × 10^4^ and treated with concentrations of 1, 2.5, 5, 12.5, 25, 50, and 100 μg/mL. After incubation for 24 h, 3-(4,5-Dimethylthiazol-2-yl)-2,5-diphenyltetrazolium bromide (MTT) reagent (at a concentration of 5 mg/mL; Duchefa Biochemie, Haarlem, The Netherlands) was added, and the cells were incubated at 37 °C in 5% CO_2_ for 2 h. Subsequently, 100 μL of 100% dimethyl sulfoxide was added to dissolve the resulting formazan crystals, and the absorbance was measured at 570 nm [20]. After seeding the cells, IL-1β was added at a concentration of 50 ng/mL, and the sample was incubated for 24 h.

### 2.6. Western Bolt

CHON-001 cells were seeded in 6-well plates at a density of 3 × 10^5^ cells/mL and incubated for 24 h at 37 °C. After pretreatment with 0, 12.5, 25, or 50 μg/mL DMWE for 12 h, the cells were stimulated with 50 ng/mL IL-1β for 24 h at 37 °C. Total protein was extracted by adding lysis buffer (Millipore Sigma, Burlington, MA, USA) supplemented with 1X Inhibitor cocktail solution (#P3300-005, Gendepot, Altair, TX, USA), placing the cells on ice for 10 min and then centrifuging for 15 min at 12,000 rpm at 4 °C. Protein concentration was quantified using an enhanced chemiluminescence protein assay kit (Beyotime Institute of Biotechnology, Shanghai, China), and 20 µg protein/well was separated via 8%.

The separated proteins were subsequently transferred onto 0.2 µm polyvinylidene fluoride membranes (Bio-Rad Laboratories, Inc., Hercules, CA, USA) and blocked with 5% skimmed milk for 2 h at room temperature. The membranes were then placed in 5% bovine serum albumin or Fraction V and incubated with the following primary antibodies overnight at 4 °C: Cyclooxygenase-2 (COX-2, 1:1000), Inducible Nitric Oxide Synthase (iNOS, 1:1000), MMP-2 (1:1000), MMP-8 (1:1000), Collagen Type II Alpha 1 Chain (COL2A1, 1:1000), p-JAK2 (1:1000), and p-STAT1 (1:1000). Following the primary antibody incubation and washing with Tris-buffered saline (Beyotime Institute of Biotechnology) with 0.1% Tween 20, the membranes were incubated with secondary antibodies (1:5000) for 2 h at room temperature. Protein bands were visualized using an enhanced chemiluminescence solution (Millipore Sigma) and were semi-quantified using Image Lab 3.0 software (Bio-Rad Laboratories, Inc.) [21,22]. β-actin was used as the loading control.

### 2.7. Animal

Specific pathogen-free (SPF) female Balb/c mice, aged 6 weeks (weight, 15.54 ± 0.88 g), were procured from Samtaco Korea (Osan, Republic of Korea). Prior to their use in the experiments, microbiological testing was conducted on all experimental animals to confirm the absence of pathogens, thereby validating their SPF status. The animals were randomly placed in groups of 8 in cages and allowed to acclimate to their environment for one week. Bedding, feed, and drinking water were sterilized via autoclaving to maintain SPF conditions, and both water and food were provided ad libitum. The ambient temperature was maintained at 22 ± 1 °C, while humidity was controlled at 50 ± 10%. The ventilation rate was adjusted automatically to achieve 10–20 exchanges per hour, and a 12 h light/dark cycle was established (lights on: 7:00, lights off: 19:00) for the housing of the experimental animals (Three-Shine Inc., Daejeon, Republic of Korea). All animal experiments were approved by the Laboratory Animal Ethics Committee of the Daegu-Gyeongbuk Advanced Medical Industry Promotion Foundation and conducted in accordance with its regulations (Ethical Protocol Code: KMEDI-22092301-00 and Approval Date: 23 September 2022).

### 2.8. Animal Experiment Design

**Figure 1:**
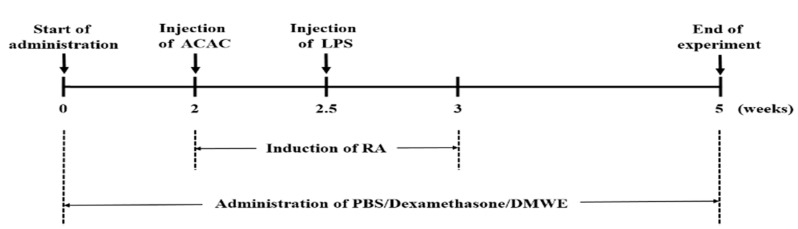
Experimental timeline for induction and treatment of RA in an animal model.

To ensure an even distribution of body weight across the test groups, each group consisted of 8 animals, making up 6 experimental groups: negative control (NC), the collagen antibody-induced arthritis (CAIA) and lipopolysaccharide (LPS)-induced model group (PC), the dexamethasone-treated group (DEXA), and the DMWE-treated groups (DMWE-1, DMWE-2, and DMWE-3). RA was induced in all groups of mice, except in the NC group, following a previously described method [23]. Specifically, mice were intraperitoneally injected once with 1.5 mg of an anti-collagen II antibody cocktail (Miceanti-type II collagen 5-clone monoclonal antibody cocktail kit, Chondrex, Redmond, WA, USA). Four days later, an intraperitoneal injection of 25 mg lipopolysaccharide (LPS, Chondrex, Redmond, WA, USA) was administered to induce RA [24]. The RA induction and drug administration schedule are depicted in Figure 1. For the DEXA group, we administered intraperitoneally at 0.5 mg/kg body weight (BW) once daily for 5 weeks, starting 2 weeks before RA induction and continuing for 3 weeks after induction, as referenced in a previous study [25]. For the DMWE groups (DMWE-1, DMWE-2, DMWE-3), DMWE was administered orally at doses of 125, 250, and 500 mg/kg BW, respectively, once daily at the same time each day for 5 weeks, starting 2 weeks prior to RA induction and continuing 3 weeks after induction. The NC and PC groups received phosphate-buffered saline (PBS) orally once daily during the same period.

### 2.9. Enzyme-Linked Immunosorbent Assay (ELISA)

Using the serum obtained from animals, the concentrations of the inflammation-related cytokines TNF-α and IL-6 were measured using a Mouse TNF-α ELISA kit (Sigma-Aldrich, St. Louis, MO, USA) and Mouse IL-6 ELISA kit (Sigma-Aldrich, St. Louis, MO, USA) [26]. The experimental process was performed by referring to the manufacturer’s instructions.

### 2.10. Scoring of Inflammation Degree

After administering the medication for 5 weeks, starting 2 weeks before RA induction and continuing until 3 weeks after RA induction, clinical observations were conducted visually based on previously described methods [25,27]. The severity of the clinical findings was scored on a scale from 0 to 4 according to the criteria outlined in Table 1.

### 2.11. Micro-CT Imaging

After the experiment was completed, all of the experimental animals were sacrificed, and the hind limbs were separated for each group. Then, before rigor mortis occurred, the toes were stretched as much as possible and fixed in a 10% formaldehyde solution. Next, the entire hind limb was scanned for 4.5 min using high-resolution micro-CT (Quantum FX micro-CT Imaging System, PerkinElmer, Hopkinton, MA, USA) at an acceleration potential of 90 kVp and a beam current of 180 µA. The field of view (FOV) was 40 mm, and the voxel size was 78.125 µm. Considering the impact of resolution, the best possible image parameters were obtained by adjusting the FOV. For one scan, the total X-ray dose was set to 1100 mGy, and the micro-CT data were reconstructed and shaped using a software program (OnDemand 3DTM, Cybermed, Seoul, Republic of Korea) and then analyzed. Using the Micro-CT software program, version 1.15. the three-dimensional microstructure of the ankle and toe bones of the left hind limb was analyzed to evaluate bone volume (BV), total volume (TV), and BV/TV ratio [28].

### 2.12. Hematoxylin and Eosin (H&E) Staning

Ankle joint tissues were harvested and fixed in 4% paraformaldehyde, followed by decalcification with 10% neutral buffered ethylenediaminetetraacetic acid. The tissues were obtained, fixed, decalcified, embedded in paraffin, and sectioned at a thickness of 5 µm [29]. The joint sections underwent deparaffinization using xylene and were dehydrated in a gradient ethanol series. The sections were stained with hematoxylin for 5 min, differentiated with 1% hydrochloric acid ethanol for 30 s, treated with 0.2% ammonia for bluing, and stained with 0.5% eosin for 10 min. Finally, the sections were observed under a light microscope.

### 2.13. Statistical Analysis

Data are expressed as mean ± standard error of the mean (SEM). Statistical analyses were conducted using one-way analysis of variance (ANOVA) followed by Duncan’s multiple-range test and the Kruskal–Wallis test for post hoc comparisons using the Statistical Package for the Social Sciences version 20 (IBM, New York, NY, USA). A *p*-value of less than 0.05 was considered statistically significant.

## 3. Results

### 3.1. Analysis of Principal Components of DMWE

**Figure 2:**
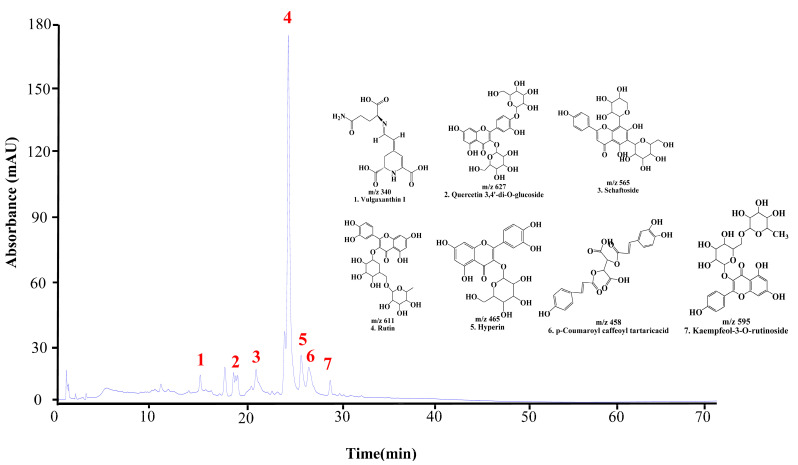
UPLC chromatogram of DMWE. Vulgaxanthin I (1), quercetin 3,4′-di-o-glucoside (2), schaftoside (3), Rutin (4), hyperin (5), caffeoyltartaric-p-coumaroyl acid (6), kaempferol-3-O-rutinoside (7).

The UPLC chromatogram of DMWE displays the presence of multiple bioactive compounds. The peaks were identified as eight compounds: vulgaxanthin I, quercetin 3,4′-di-o-glucoside, schaftoside, Rutin, hyperin, caffeoyltartaric-p-coumaroyl acid, and kaempferol-3-o-rutinoside (Figure 2). Rutin is acknowledged as the primary indicator compound of DMWE. Additionally, Table 2 and Figure S2 illustrate the fragmentation pattern of eight molecular structures, offering valuable information on the chemical composition of DMWE.

### 3.2. Antioxidant Activity in DMWE

Table 3 presents the results of the DPPH and ABTS radical scavenging activities. At DMWE concentrations of 1, 5, 10, and 50 mg/mL, the DPPH radical scavenging activities were 6.54 ± 0.48%, 20.13 ± 2.41%, 43.20 ± 5.89%, and 75.11 ± 3.08%, respectively. The positive control, vitamin C (91.64 ± 0.75%), showed statistically significantly higher scavenging activity at all concentrations of DMWE (*p* < 0.05). However, the DPPH radical scavenging activity of DMWE increased in a concentration-dependent manner, with a statistically significant difference in DPPH radical scavenging activity between the DMWE-treated groups (*p* < 0.05). At DMWE concentrations of 1, 5, 10, and 50 mg/mL, the ABTS radical scavenging activities were 6.98 ± 1.73%, 24.37 ± 2.22%, 40.13 ± 4.31%, and 63.69 ± 5.12%, respectively. The positive control, vitamin C (92.49 ± 1.22%), exhibited statistically significantly higher scavenging activity at all concentrations of DMWE (*p* < 0.05). Similarly to the DPPH results, the ABTS radical scavenging activity of DMWE increased in a concentration-dependent manner, with a statistically significant difference in ABTS radical scavenging activity between the DMWE-treated groups (*p* < 0.05).

### 3.3. Molecular Docking Study of Rutin as a Potential MMP-8 Inhibitor for RA Therapy

**Figure 3:**
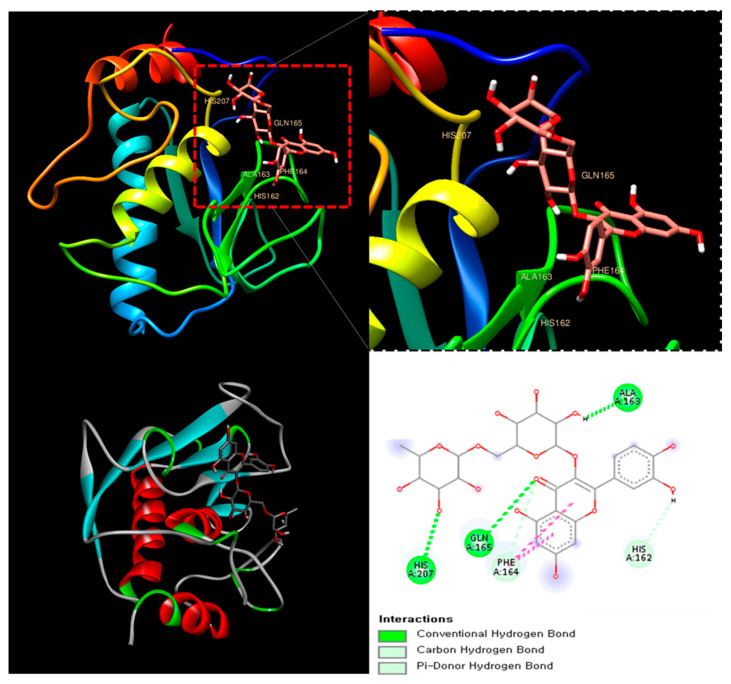
The catalytic domain of MMP-8 (PDB ID: 1BZS) with Rutin at the binding site.

The molecular docking analysis of Rutin with MMP-8 (PDB ID: 1BZS) revealed a binding affinity score of −7.7 kcal/mol, suggesting a strong interaction. Rutin formed multiple hydrogen bonds with key amino acid residues, including Histidine 162, Glutamine 165, and Alanine 163, stabilizing its interaction within the active site of MMP-8. Additionally, π-stacking interactions were observed, further enhancing ligand binding (Figure 3). MMP-8 plays a crucial role in the degradation of extracellular matrix components, contributing to joint damage and inflammation in RA [30].

This result suggests that Rutin could serve as a natural MMP-8 inhibitor, potentially mitigating the progression of RA through its anti-inflammatory and chondroprotective properties.

### 3.4. DMWE Downregulates the IL-1β-Induced Upregulation of Inflammation Expression in CHON-001 Cells

**Figure 4:**
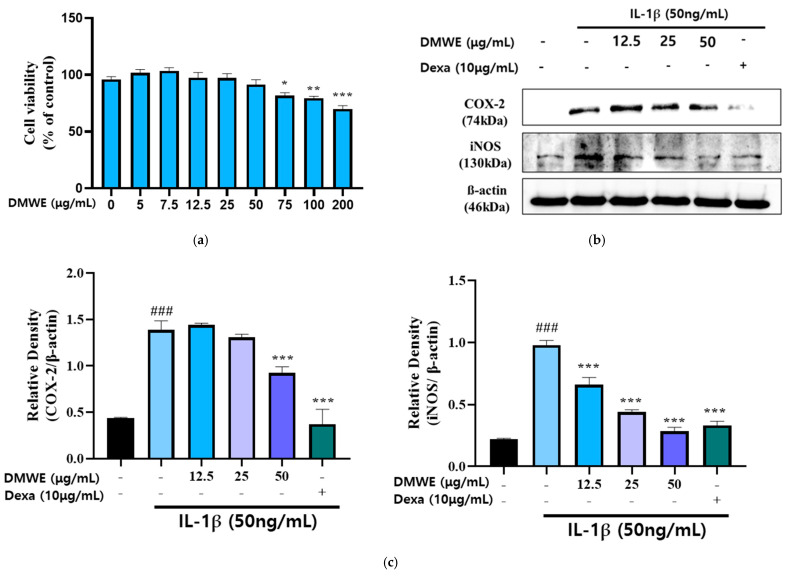
Effects of DMWE on IL-1β-induced inflammation activation in CHON-001 cell. (**a**) Cell toxicity in CHON-001 Cells. Data represents the mean ± SEM of three independent experiments. (**b**) The protein expression levels were determined by Western blot with β-actin as the internal control (**c**) quantification analysis. Data represent the mean ± SEM. ^###^ *p* < 0.001 vs. untreated control. * *p* < 0.05, ** *p* < 0.01, *** *p* <  0.001 vs. IL-1β-treated group.

We treated the chondrocytes with DMWE at different concentrations (0~200 μg/mL) for 24 h, respectively. The MTT assay indicated that chondrocyte viability was non-significantly reduced after treatment with 12.5, 25, and 50 μg/mL DMWE for 24 h (Figure 4a), respectively, indicating that DMWE at ≤50 μg/mL exhibited no obvious cytotoxicity to chondrocytes. Hence, we selected DMWE at 12.5, 25, and 50 μg/mL to explore its role in RA progression. In this study, the impact of DMWE on IL-1β-induced chondrocyte inflammation was examined through treatments at concentrations of 12.5, 25, and 50 μg/mL. The findings indicated a dose-dependent inhibitory effect of DMWE on iNOS expression. Notably, the most substantial reduction in COX-2 expression was observed at the highest concentration of 50 μg/mL DMWE. The positive control at a concentration of 10 μg/mL demonstrated greater effectiveness in comparison to the extract (Figure 4b,c).

### 3.5. DMWE Downregulates the IL-1β-Induced Upregulation of MMPs Expression in CHON-001 Cells

**Figure 5:**
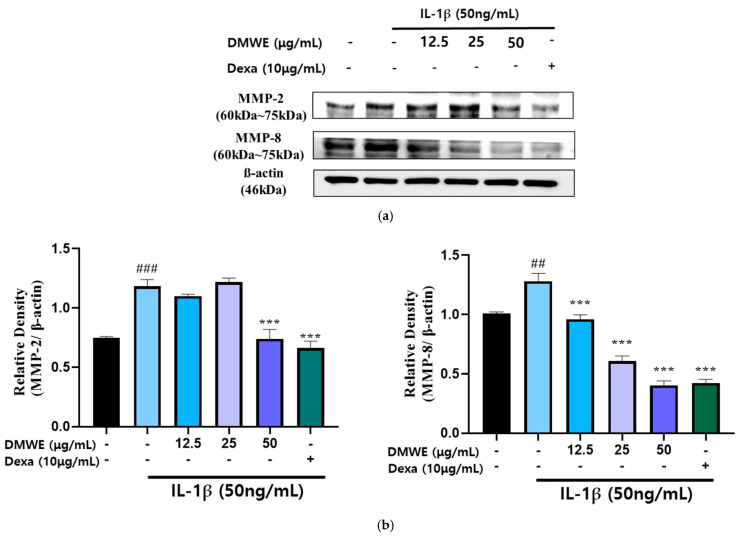
Effect of DMWE on the expression of MMP-2 and MMP-8 in CHON-001 Cells. (**a**) The protein expression levels MMP-2 and MMP-8 were determined by Western blot with β-actin as the internal control. (**b**) MMPs quantification analysis. Data represents the mean ± SEM. ^##^ *p* < 0.01, ^###^ *p* < 0.001 vs. untreated control. *** *p* < 0.001 vs. IL-1β -induced group.

RA is a persistent inflammatory condition marked by the increased production of inflammatory cytokines and MMPs, leading to cartilage deterioration and joint injury. In order to assess the impact of DMWE on IL-1β-induced MMP expression, CHON-001 cells were exposed to IL-1β at a concentration of 50 ng/mL in the presence or absence of DMWE at varying concentrations (12.5, 25, and 50 μg/mL). Dexa at a concentration of 10 μg/mL was used. The Western blot analysis demonstrated that stimulation with IL-1β resulted in a notable increase in the expression of MMP-2 and MMP-8, suggesting their role in extracellular matrix degradation and inflammatory pathways in RA. However, the administration of DMWE resulted in a dose-dependent reduction in IL-1β-induced MMP-2 and MMP-8 expression levels, indicating that DMWE may exert anti-inflammatory and cartilage-protective effects. In addition, the inhibitory effects of DMWE were similar to those of Dexa (Figure 5), a widely recognized anti-inflammatory medication, indicating its potential usefulness in treating RA.

### 3.6. DMWE Downregulates the IL-1β-Induced Upregulation of JAK2/STAT Pathway Expression in CHON-001 Cells

**Figure 6:**
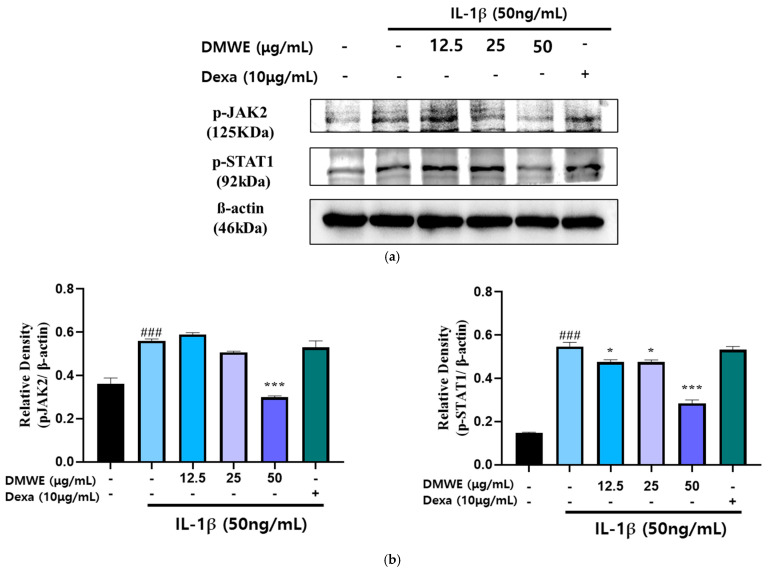
Effect of DMWE on the expression of JAK2/STAT in CHON-001 Cells. (**a**) The protein expression levels were determined by Western blot with β-actin as the internal control. (**b**) p-JAK2 and p-STAT1 quantification analysis. Data represents the mean ± SEM. ^###^ *p* < 0.001 vs. untreated control. * *p* < 0.05; *** *p* < 0.001 vs. IL-1β-induced group.

To assess the suppressive impact of DMWE on the IL-1β-induced activation of the JAK2/STAT signaling pathway, CHON-001 cells were exposed to IL-1β (50 ng/mL) with or without DMWE (12.5, 25, and 50 μg/mL). Dexa at a concentration of 10 μg/mL was used. The analysis using Western blotting indicated that stimulation with IL-1β led to a significant elevation in the phosphorylation levels of JAK2 and STAT1 in comparison to the untreated control. However, treatment with DMWE at a concentration of 50 μg/mL significantly inhibited the IL-1β-induced phosphorylation of JAK2 and STAT1, suggesting its ability to suppress inflammatory signaling in cartilage cells (Figure 6). These findings indicate that DMWE could potentially serve as a therapeutic intervention for decreasing inflammatory responses by modulating the JAK2/STAT pathway.

### 3.7. Changing of Body Weights

**Figure 7:**
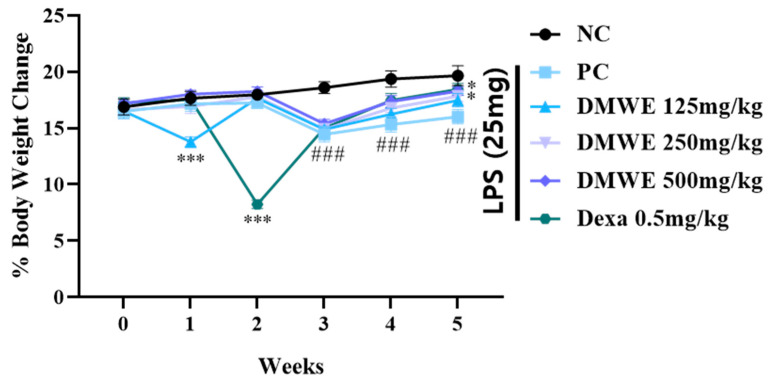
Changes in body weight as a percentage of DMWE in RA mice models. Statistical analysis was performed according to a two-way ANOVA with Dunnett’s multiple comparison test. n = 8 for each group. ^###^ *p* < 0.001 vs. negative control. * *p* < 0.05; *** *p* < 0.001 versus RA-induced mouse model.

Figure 7 depicts the alterations in body weight noted across all groups from two weeks before the onset of RA to three weeks after induction. Within the first two weeks after the commencement of drug treatment, there were no notable discrepancies in body weight among any of the groups. During the third week following the induction of RA, there was a noticeable decrease in body weight within all experimental groups when compared to the NC group, with statistical significance. Nevertheless, there were no notable differences observed between the groups treated with DEXA and DMWE. During week four post drug administration, all treatment groups exhibited significantly lower body weights compared to the NC group. In contrast, the body weights of the groups DEXA, DMWE-2, and DMWE-3 shows a significant increase when compared to the PC group. By the fifth week post drug treatment, a notable and significant rise in body weight was observed across all treatment groups compared to the PC group. Additionally, there were no significant differences in body weights between the DEXA, DMWE-2, and DMWE-3 groups when compared to the NC group.

### 3.8. Anti-Inflammatory Effects of DMWE on Serum TNF-α and IL-6 in RA Mouse Models

**Figure 8:**
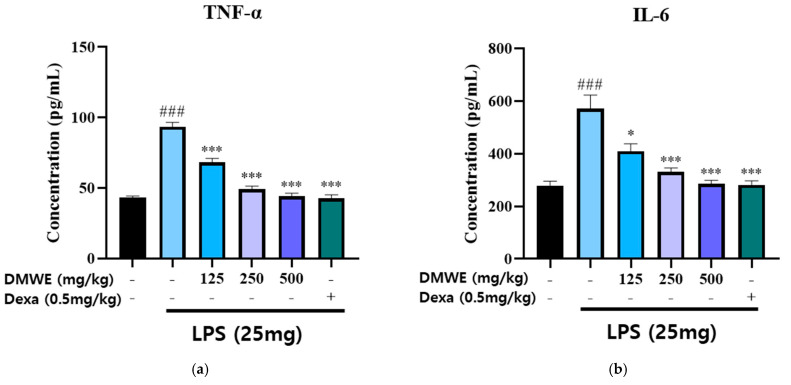
Effects of DMWE on pro-inflammatory cytokine levels in serum in RA mice model. (**a**) The serum levels of TNF-α and (**b**) IL-6 were detected using an ELISA. Data represents the mean ± SEM. n = 8 for each group. ^###^ *p* < 0.001 vs. negative control. * *p* < 0.05; *** *p* < 0.001 vs. RA-induced mouse model.

The levels of TNF-α and IL-6 in the serum were measured in blood samples collected from all animals in the study, starting two weeks before and lasting three weeks after the induction of RA following drug administration. As shown in Figure 8a, the concentration of TNF-α in the LPS-induced group was markedly increased compared to the NC group. Treatment with DMWE at doses of 125, 250, and 500 mg/kg significantly reduced TNF-α levels in a dose-dependent manner. The reduction observed at the highest dose (500 mg/kg) was comparable to that of dexamethasone (0.5 mg/kg). Similarly, Figure 8b shows that the IL-6 levels were significantly increased in the LPS-induced group compared to the NC group. Treatment with DMWE at doses of 125, 250, and 500 mg/kg led to a marked decrease in IL-6 levels, demonstrating a dose-dependent anti-inflammatory effect. Dexamethasone administration also significantly reduced IL-6 levels. These findings suggest that DMWE effectively attenuates the inflammatory response induced by LPS by reducing the serum levels of the pro-inflammatory cytokines TNF-α and IL-6.

### 3.9. Effects of DMWE on Hind Limb Edema in RA Mouse Models

**Figure 9:**
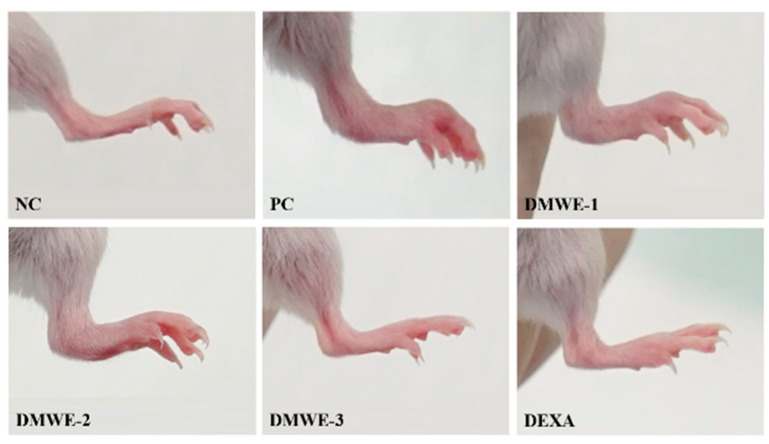
Representative macroscopic photographs of Balb/C mice models. Negative control group (NC); RA-induced model group (PC); DMWE-treated groups (DMWE-1, DMWE-2, and DMWE-3); Dexametasone (DEXA).

Images of the hind limbs in mice with experimentally induced RA after a 5-week treatment regimen are shown in Figure 9. All groups that were given DMWE demonstrated a dose-dependent reduction in symptoms compared to the PC group. Remarkably, DMWE-3 exhibited a level of enhancement comparable to that observed with DEXA, showing no substantial deviation from the NC (Figure 9). Table 4 delineates the edema scores observed in the hind limbs induced with RA after a 5-week treatment regimen. The edema scores in each treatment group exhibited a notable decrease in comparison to the PC group with a statistical significance level of *p* < 0.05. There were no statistically significant differences observed between the groups receiving treatment with DMWE and the DEXA group, as well as among the various groups treated with DMWE.

### 3.10. A Micro-CT Study on the Impact of DMWE on Bone Structure in RA Mouse Models

**Figure 10:**
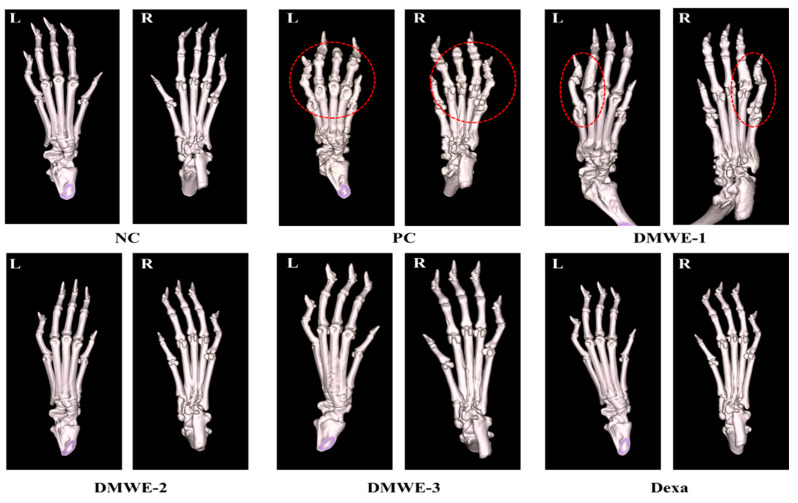
Micro-CT scanning of hind paws of mice from different groups. L: left, R: right. Red dotted circle: observation of deformation around joints.

Figure 10 depicts micro-CT images of the left and right hind limbs acquired after a 5-week treatment regimen subsequent to RA induction. The NC group displayed typical anatomical structures in both the left and right hind limbs, showing no signs of abnormalities. On the other hand, the PC group showed notable abnormalities near the joints of the hind limbs, as marked by red encircling, which can be attributed to RA. The DEXA, DMWE-2, and DMWE-3 cohorts exhibited typical joint morphology, free from any deformities induced by RA in the vicinity of the joints. In the DMWE-1 group, instances of joint deformities were noted in specific areas surrounding the joints (highlighted by red circles) as a result of RA, while other joint areas appeared unaffected. Table 5 displays the bone indices obtained using micro-CT analysis. The BV of the DEXA, DMWE-2, and DMWE-3 groups exhibited a statistically significant increase when compared to the PC group, whereas the BV of the DMWE-1 group did not demonstrate a significant difference. Additionally, there were no notable variances in BV between the groups that received DMWE treatment. The BV fraction of the DEXA, DMWE-2, and DMWE-3 groups exhibited a statistically significant increase compared to the PC group, whereas that of the DMWE-1 group did not differ significantly. In the same vein, there were no significant variations in BV/TV noted across the DMWE-treated groups.

### 3.11. Histopathological Analysis of Joint Tissues in RA Mouse Models

**Figure 11:**
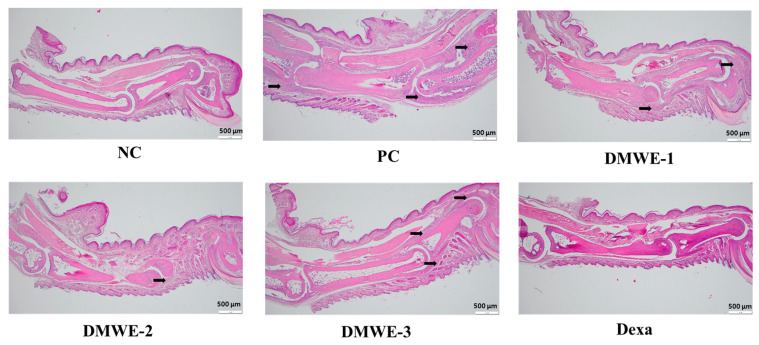
Histological analysis of joint tissues in the RA animal model treated with DMWE: RA representative symptoms, Arrow: This location marks the area of lesion change, scale bar = 500 μm.

The NC group exhibits normal joint architecture without signs of inflammation, synovial hyperplasia, or cartilage erosion. The PC group shows severe synovial hyperplasia, inflammatory cell infiltration, cartilage destruction, and pannus formation, which are characteristic of RA pathology. DMWE-1 partially reduces synovial hyperplasia and inflammatory cell infiltration, although cartilage damage remains. The efficacy of DMWE in alleviating RA symptoms is dose-dependent, with the DMWE-3 yielding results similar to those of dexamethasone. DMWE-1 and DMWE-2 offer partial protection, but are less effective in reversing cartilage damage. At higher doses, DMWE demonstrates potent anti-inflammatory and chondroprotective effects, supporting the hypothesis that it could serve as a viable alternative to traditional RA treatments (Figure 11).

## 4. Discussion

RA is an autoimmune disorder that emphasizes the importance of timely diagnosis and intervention [31]. At present, there is a lack of specific curative treatments for RA in clinical practice, with pharmacotherapy being the primary therapeutic approach. Treatment goals involve reducing pain, managing inflammatory processes, slowing down the progression of the disease, and preserving joint function as much as possible. Recent research has suggested that specific natural compounds could potentially assist in the management of RA by helping to reduce inflammation, relieve pain, and possibly enhance joint health [32,33]. This study examines the therapeutic benefits of DMWE for RA, specifically in terms of its anti-inflammatory and antioxidant properties. The analysis conducted using DMWE UPLC-TOF-MS/MS resulted in the identification of seven distinct peaks. Rutin, a flavonoid glycoside with the chemical formula C_27_H_30_O_16_, is frequently present in a variety of plants, particularly citrus fruits, apples, and buckwheat [34]. Several studies have explored its anti-inflammatory, antioxidant [35], and potential therapeutic properties in addressing conditions such as cardiovascular disease and diabetes [36]. In the current investigation, Rutin was identified with a molecular weight of 611 *m*/*z*, and its fragmentation pattern in the MS/MS spectrum revealed a prominent ion at 303 *m*/*z*. This indicates that structural features, such as the flavonoid backbone and sugar components, play a role in its biological function (Figure S2 and Table 2) [37]. DMWE, similar to other flavonoids, suggests various biological effects through interactions with signaling pathways, including notable antioxidants and anti-inflammatory activities that have implications for the management of chronic diseases such as RA and other inflammatory conditions.

DMWE exhibited notable antioxidant properties by effectively scavenging DPPH and ABTS radicals in a manner that correlated with the dosage administered (Table 3). The antioxidant properties of these substances are essential for reducing oxidative stress, a key factor in the development of RA. In vitro experiments additionally demonstrated that DMWE effectively decreased the expression of pro-inflammatory enzymes. This inhibitory effect suggests a potential mechanism by which DMWE could potentially prevent inflammation, specifically targeting enzymes such as COX-2 and iNOS in chondrocytes exposed to IL-1β (Figure 4).

RA is a persistent inflammatory disorder identified by the heightened production of pro-inflammatory cytokines and MMPs [38], which lead to the breakdown of the extracellular matrix and consequent damage to the joints. During RA, increased levels of MMP-2 [39,40] and MMP-8 [30,41] are crucial in the degradation of the extracellular matrix, resulting in the destruction of cartilage and inflammation within the impacted joints. In this investigation, we assessed the impact of the effects of DMWE on IL-1β-induced MMP expressions that were studied in CHON-001 cells, a chondrocyte model. The findings indicate that IL-1β, a pro-inflammatory cytokine commonly linked to the development of RA, notably elevated the expression of MMP-2 and MMP-8 in CHON-001 cells, as validated using Western blot analysis. This observation highlights the significant involvement of MMPs in inflammation and cartilage degradation evident in RA. Significantly, the administration of DMWE led to a reduction in IL-1β-induced MMP-2 and MMP-8 expression in a manner dependent on dosage, indicating the anti-inflammatory and chondroprotective attributes of DMWE (Figure 5). The inhibitory effect of DMWE was comparable to that of dexamethasone, a well-known anti-inflammatory drug, indicating that DMWE may offer similar therapeutic benefits in the treatment of RA.

In RA, the upregulation of MMP-8 plays a pivotal role in extracellular matrix degradation, leading to cartilage breakdown and inflammation in the affected joints. Further analysis of the potential of Rutin, a marker compound known to be present in DMWE, as a natural MMP-8 inhibitor via molecular docking studies, revealed a binding affinity score of −7.7 kcal/mol (Figure 3).

Current management protocols recommend prompt and proactive treatment in order to attain the objectives of minimizing disease activity or achieving remission expeditiously. As a result, RA is presently treated using a wide range of therapeutic agents, including steroidal/non-steroidal anti-inflammatory drugs and glucocorticoids, as well as synthetic disease-modifying anti-rheumatic drugs of synthetic origin. These disease-modifying anti-rheumatic drugs (DMARDs) consist of conventional synthetic methotrexate, biological and biosimilar TNF-α inhibitors, or IL-6 inhibitors and targeted synthetic JAK2 inhibitors, which purposefully target specific immune cells, cytokines, or inflammatory pathways [42,43].

This study also highlights DMWE’s ability to modulate the JAK2/STAT signaling pathway, a key mediator in inflammatory processes. By inhibiting the phosphorylation of JAK2 and STAT1, DMWE reduces the inflammatory response, providing further evidence of its therapeutic potential in RA (Figure 6). The suppression of this pathway is crucial, as it is often upregulated in inflammatory conditions and contributes to the persistence of inflammation and joint destruction.

In RA, heightened vascular permeability, degradation of the extracellular matrix, and infiltration of immune cells into the synovial tissue lead to the transformation of the normally low-cellularity synovium into persistently inflamed tissue [44]. This process includes the enlargement of the synovial lining, along with the stimulation of macrophages and fibroblast-like synoviocytes [45]. Subsequently, these cells secrete various inflammatory mediators, including IL-1β, IL-6, IL-8, TNF-α, granulocyte–macrophage colony-stimulating factor, macrophage migration inhibitory factor, matrix-degrading enzymes such as MMPs, and a disintegrin and metalloproteinase with thrombospondin motifs, as well as prostaglandins, leukotrienes, and reactive nitric oxide species [46]. The extended activated fibroblast-like synoviocytes play a role in cartilage degradation by developing hyperplastic pannus tissue. Additionally, these cells have the capability to migrate between joints and other organs, potentially spreading inflammation throughout the body [43,47,48].

In vivo experiments using RA-induced mouse models showed that the oral administration of DMWE led to a significant reduction in joint swelling and improvements in body weight and joint morphology (Figure 7 and Figure 9). Notably, higher doses of DMWE exhibited effects similar to those of dexamethasone, a standard anti-inflammatory drug used in RA treatment. This suggests that DMWE may offer a viable alternative to traditional pharmacological treatments, potentially reducing the reliance on synthetic drugs and their associated side effects. In addition, we investigated the anti-inflammatory effects of DMWE on pro-inflammatory cytokines TNF-α and IL-6 in an RA mouse model induced by LPS administration. Our results demonstrate that DMWE treatment significantly reduced the serum levels of both TNF-α and IL-6 in a dose-dependent manner, comparable to the effects observed with dexamethasone (Figure 8).

TNF-α and IL-6 are key pro-inflammatory cytokines involved in the pathogenesis of RA, contributing to the activation and maintenance of chronic inflammation within the joint microenvironment. Increased levels of these cytokines contribute to tissue damage, the infiltration of immune cells, and the sustained activation of inflammatory responses. Therefore, the suppression of TNF-α and IL-6 levels is considered an important therapeutic target for the management of RA.

Micro-CT imaging further supported these findings by demonstrating preserved bone structure and reduced joint deformities in DMWE-treated groups (Figure 10). A histopathological analysis confirmed the protective effects of DMWE on joint tissues, with higher doses effectively reducing synovial hyperplasia and inflammatory cell infiltration (Figure 11). These results underscore the potential of DMWE in preventing the progression of RA and preserving joint integrity.

This study provides compelling evidence that DMWE possesses significant antioxidant and anti-inflammatory properties, making it a promising candidate for RA therapy. Its ability to modulate key inflammatory pathways and protect joint tissues suggests that DMWE could be developed into a natural therapeutic agent for RA. Future research should focus on elucidating the detailed molecular mechanisms underlying its effects and conducting clinical trials to confirm its efficacy and safety in humans. Additionally, exploring the synergistic effects of DMWE with existing RA treatments could further enhance its therapeutic potential.

## 5. Conclusions

This study confirms the excellent antioxidant and anti-inflammatory effects of DMWE. These effects prevented the destruction of bone and cartilage caused by chronic inflammation in RA-induced mice, showing a strong therapeutic effect on RA. Therefore, the findings indicate that DMWE (300 mg/kg BW) can be used as a natural auxiliary treatment for RA.

## Figures and Tables

**Table 1:** Grading scale for inflammation and edema in an arthritis model.

Grade	Clinical Findings
0	Normal appearance, no signs of inflammation
1	Inflammation and edema present in one toe
2	Inflammation and edema present in one or more toes, but not involving the entire foot
3	Inflammation and edema involving the entire foot
4	Joint stiffness and generalized inflammation throughout the foot

**Table 2:** Date for characterization of compounds in DMWE by UPLC-TOF-MS/MS.

Peak No.	Retention Time (min)	Formula	Identification	Experiment Mass (*m*/*z*)	MS/MS
1	15.73	C_14_H_17_N_3_O_7_	Vulgaxanthin I	340	322, 209
2	18.99	C_27_H_30_O_17_	Quercetin 3,4′-di-O-glucoside	627	465, 303
3	21.19	C_26_H_28_O_14_	Schaftoside	565	547, 445, 325
4	23.98	C_27_H_30_O_16_	Rutin	611	303
5	25.59	C_21_H_20_O_12_	Hyperin	465	303
6	26.36	C_22_H_18_O_11_	p-Coumaroyl caffeoyl tartaric acid	458	295, 277
7	28.41	C_27_H_30_O_15_	Kaempferol-3-O-rutinoside	595	287

**Table 3:** DPPH and ABTS radical scavenging activities of DMWE.

Groups *	Concentration (mg/kg)	DPPH Radical Scavenging Activity (%)	ABTS Radical Scavenging Activity (%)
Control	1	91.64 ± 0.75 ^a^	92.49 ± 1.22 ^a^
DMWE	1	6.54 ± 0.48 ^b^	6.98 ± 1.73 ^b^
	5	20.13 ± 2.41 ^c^	24.37 ± 2.22 ^c^
	10	43.20 ± 5.89 ^d^	40.13 ± 4.31 ^d^
	50	75.11 ± 3.08 ^e^	63.69 ± 5.12 ^e^

* Control: vitamin C 1 mg/kg; DMWE. The different superscript letters at the same column indicate statistically significant difference (a, b, c, d, e, *p* < 0.05).

**Table 4:** Critical arthritis index score.

Groups	Score
NC	0.00 ± 0.00 ^a^
PC	3.80 ± 0.42 ^b^
DEXA	1.20 ± 0.42 ^c^
DMWE-1	1.80 ± 0.79 ^cd^
DMWE-2	1.40 ± 0.52 ^cd^
DMWE-3	1.20 ± 0.42 ^cd^

The different superscript letters indicate statistically significant differences (a, b, c, d, *p* < 0.05).

**Table 5:** Bone morphological microstructure of bone of hind paws in mice induced RA after administration of DMWE.

Groups	Bonindex		
	BV (mm^3^)	TV (mm^3^)	BV/TV (%)
NC	0.37 ± 0.01 ^a^	1.29 ± 0.00	28.91 ± 0.90 ^a^
PC	0.27± 0.05 ^b^	1.29 ± 0.00	20.54 ± 3.69 ^b^
DEXA	0.34 ± 0.03 ^c^	1.29 ± 0.00	26.45 ± 2.70 ^c^
DMWE-1	0.28 ± 0.05 ^bd^	1.29 ± 0.00	21.37 ± 4.05 ^bd^
DMWE-2	0.31 ± 0.07 ^cd^	1.29 ± 0.00	24.03 ± 5.43 ^cd^
DMWE-3	0.33 ± 0.04 ^cd^	1.29 ± 0.00	25.36 ± 2.75 ^cd^

The different superscript letters at the same column indicate statistically significant differences (a, b, c, d, *p* < 0.05). Bone volume (BV), total volume (TV).

## Data Availability

The original contributions presented in this study are included in the article and Supplementary Materials. Further inquiries can be directed to the corresponding author.

## Supplementary Materials

The following supporting information can be downloaded at: https://www.mdpi.com/article/10.3390/antiox14050548/s1. Figure S1: Flow diagram for the preparation of *Dendropanax morbiferus Lév.* leaf water extracts. Figure S2: Fragmentation scheme of the compounds identified from the DMWE by the UPLC-TOF/MS analysis.

## Abbreviations

The following abbreviations are used in this manuscript:

ABTS	2,2-azino-bis(3-ethylbenzothiazoline-6-sulfonic acid) diammonium salt
ACAC	Anti-collagen II antibody cocktail
ANOVA	Analysis of variance
BCA	Bicinchoninic acid
BV	Bone volume
BW	Body weight
CAIA	Collagen Antibody-Induced Arthritis
COX-2	Cyclooxygenase-2
COL2A1	Collagen Type II Alpha 1 Chain
DEXA	Dexamethasone
DMWE	*Dendropanax Morbiferus Lév*. leaf water Extracts
DPPH	1,1-diphenyl-2-picrylhydrazyl
ESI	Electrospray ionization source
ELISA	Enzyme-Linked Immunosorbent Assay
FBS	Fetal bovine serum
FOV	Field of view
H&E	Hematoxylin and Eosin
IL-6	Interleukin-6
iNOS	Inducible nitric oxide synthase
JAK2	Janus Kinase 2
LPS	Lipopolysaccharide
MMPs	Matrix metalloproteinases
MTT	(3-(4,5-Dimethylthiazol-2-yl)-2,5-diphenyltetrazolium bromide)
Micro-CT	Micro-computed tomography
m/z	Mass-to-charge
NC	Negative control
PBS	Phosphate-buffered saline
PC	Positive control
QTOF/MS	Quadrupole time-of-flight/mass spectrometry
RA	Rheumatoid Arthritis
SEM	Standard Error of the Mean
SPF	Specific pathogen-free
STAT	Signal Transducer and Activator of Transcription
solution A	0.1% formic acid in H_2_O
solution B	0.1% formic acid in acetonitrile
TV	Total volume
TNF-α	Tumor necrosis factor-α
UPLC	Ultra-High-Performance Liquid Chromatography
DMARDs	Disease-modifying anti-rheumatic drugs

## References

[1] Radu A.-F., Bungau S.G. (2021). Management of rheumatoid arthritis: An overview. Cells.

[2] Guo Q., Wang Y., Xu D., Nossent J., Pavlos N.J., Xu J. (2018). Rheumatoid arthritis: Pathological mechanisms and modern pharmacologic therapies. Bone Res..

[3] Kay J., Calabrese L. (2004). The role of interleukin-1 in the pathogenesis of rheumatoid arthritis. Rheumatology.

[4] Zhao X., Kim Y.-R., Min Y., Zhao Y., Do K., Son Y.-O. (2021). Natural plant extracts and compounds for rheumatoid arthritis therapy. Medicina.

[5] Mateen S., Zafar A., Moin S., Khan A.Q., Zubair S. (2016). Understanding the role of cytokines in the pathogenesis of rheumatoid arthritis. Clin. Chim. Acta.

[6] Xu T., Liu S., Zhao J., Feng G., Pi Z., Song F., Liu Z. (2015). A study on the effective substance of the Wu-tou formula based on the metabonomic method using UPLC-Q-TOF-HDMS. Mol. Biosyst..

[7] Funk J.L., Oyarzo J.N., Frye J.B., Chen G., Lantz R.C., Jolad S.D., Sólyom A.M., Timmermann B.N. (2006). Turmeric extracts containing curcuminoids prevent experimental rheumatoid arthritis. J. Nat. Prod..

[8] Kim M.J., Son J.D., Yang Y.J., Heo J.W., Lee H.J., Park K.I. (2024). LC-MS/MS analysis and antioxidant activity of Dendropanax morbiferus extract. Herb. Formula Sci..

[9] Hwang C.E., Kim S.C., Cho C.S., Song W.Y., Joo O.S., Cho K.M. (2020). Comparison of chlorogenic acid and rutin contents and antioxidant activity of Dendropanax morbiferus extracts according to ethanol concentration. Korean J. Food Preserv..

[10] Lee S.-g., Lee S.-h., Park E.-J. (2015). Antimicrobial and Antioxidant Activities of Ethanol Leaf Extract of Dendropanax morbiferus Lev. Korean J. Food Cook. Sci..

[11] Song J.-H., Kang H.-B., Kim J.H., Kwak S., Sung G.-J., Park S.-H., Jeong J.-H., Kim H., Lee J., Jun W. (2018). Antiobesity and cholesterol-lowering effects of Dendropanax morbifera water extracts in mouse 3T3-L1 Cells. J. Med. Food.

[12] Balakrishnan R., Cho D.-Y., Su-Kim I., Choi D.-K. (2020). Dendropanax morbiferus and other species from the genus Dendropanax: Therapeutic potential of its traditional uses, phytochemistry, and pharmacology. Antioxidants.

[13] Ding L., Lin H., Ma Z., He Y., Ding S., Zhang K., Zhang J., Li W., Xiao L. (2024). Stigmasterol mitigates rheumatoid arthritis progression by decreasing Nrf2/NLRP3-mediated pyroptosis in chondrocyte. Mol. Immunol..

[14] Tseng C.-C., Chen Y.-J., Chang W.-A., Tsai W.-C., Ou T.-T., Wu C.-C., Sung W.-Y., Yen J.-H., Kuo P.-L. (2020). Dual role of chondrocytes in rheumatoid arthritis: The chicken and the egg. Int. J. Mol. Sci..

[15] Kim M.J., Yang Y.J., Min G.Y., Heo J.W., Son J.D., You Y.Z., Kim H.H., Kim G.S., Lee H.J., Yang J.H. (2025). Anti-inflammatory and antioxidant properties of Camellia sinensis L. extract as a potential therapeutic for atopic dermatitis through NF-κB pathway inhibition. Sci. Rep..

[16] Gulcin İ., Alwasel S.H. (2023). DPPH radical scavenging assay. Processes.

[17] Lee S.G., Wang T., Vance T.M., Hurbert P., Kim D.-O., Koo S.I., Chun O.K. (2017). Validation of analytical methods for plasma total antioxidant capacity by comparing with urinary 8-isoprostane level. J. Microbiol. Biotechnol..

[18] Kim M.J., Yang Y.J., Heo J.W., Son J.-d., You Y.Z., Yang J.-H., Park K.I. (2025). Potential Chondroprotective Effect of Artemisia annua L. Water Extract on SW1353 Cell. Int. J. Mol. Sci..

[19] Park D.-Y., Shin W.-R., Kim S.Y., Nguyen Q.-T., Lee J.-P., Kim D.-Y., Ahn J.-Y., Kim Y.-H. (2023). In silico molecular docking validation of procalcitonin-binding aptamer and sepsis diagnosis. Mol. Cell. Toxicol..

[20] Ou D., Liu S., Tong C., Tan H., Yang Y., He C.J.E., Medicine T. (2022). LIM mineralization protein-1 inhibits IL-1β-induced human chondrocytes injury by altering the NF-κB and MAPK/JNK pathways. Exp. Ther. Med..

[21] Kim H.H., Jeong S.H., Park M.Y., Bhosale P.B., Abusaliya A., Kim H.W., Seong J.K., Ahn M., Park K.I., Heo J.D. (2023). Potential Joint Protective and Anti-Inflammatory Effects of Integrin αvβ3 in IL-1β-Treated Chondrocytes Cells. Biomedicines.

[22] Yang Y.J., Kim M.J., Heo J.W., Kim H.H., Kim G.S., Shim M.S., Kim K.Y., Park K.I. (2025). Korean Mistletoe (Viscum album var. coloratum) Ethanol Extracts Enhance Intestinal Barrier Function and Alleviate Inflammation. Antioxidants.

[23] Xie Z., Dai J., Yang A., Wu Y. (2014). A role for bradykinin in the development of anti-collagen antibody-induced arthritis. Rheumatology.

[24] Waritani T., Cutler D., Terato K. (2009). Collagen antibody-induced arthritis (CAIA) in mice: Triggering of arthritis by HMGB1, a late stage lethal mediator of LPS (99.22). J. Immunol..

[25] Inglis J.J., Criado G., Medghalchi M., Andrews M., Sandison A., Feldmann M., Williams R.O. (2007). Collagen-induced arthritis in C57BL/6 mice is associated with a robust and sustained T-cell response to type II collagen. Arthritis Res. Ther..

[26] Yang Y.J., Kim M.J., Lee H.J., Lee W.-Y., Yang J.-H., Kim H.H., Shim M.S., Heo J.W., Son J.D., Kim W.H. (2024). Ziziphus jujuba Miller Ethanol Extract Restores Disrupted Intestinal Barrier Function via Tight Junction Recovery and Reduces Inflammation. Antioxidants.

[27] Quan L., Zhang Y., Dusad A., Ren K., Purdue P.E., Goldring S.R., Wang D. (2016). The evaluation of the therapeutic efficacy and side effects of a macromolecular dexamethasone prodrug in the collagen-induced arthritis mouse model. Pharm. Res..

[28] Bouxsein M.L., Boyd S.K., Christiansen B.A., Guldberg R.E., Jepsen K.J., Müller R. (2010). Guidelines for assessment of bone microstructure in rodents using micro–computed tomography. J. Bone Miner. Res..

[29] Yang J.-H., Yoo J.-M., Cho W.-K., Ma J.Y. (2016). Ethanol Extract of Sanguisorbae Radix Inhibits Mast Cell Degranulation and Suppresses 2, 4-Dinitrochlorobenzene-Induced Atopic Dermatitis-Like Skin Lesions. Mediat. Inflamm..

[30] Mattey D.L., Nixon N.B., Dawes P.T. (2012). Association of circulating levels of MMP-8 with mortality from respiratory disease in patients with rheumatoid arthritis. Arthritis Res. Ther..

[31] Kim H.-Y., Zuo G., Lee H.J., Hwang S.H., Lee S.K., Park J.H., Suh H.-W., Lim S.S. (2024). Evaluation of the antinociceptive effect and standardization of *Platycladus orientalis* (L.) Franco extract. Mol. Cell. Toxicol..

[32] Jha L.A., Imran M., Shrestha J., Devkota H.P., Bhattacharya K., Alsayari A., Wahab S., Jha S.K., Paudel K.R., Kesharwani P. (2024). Effectiveness of phytoconstituents and potential of phyto-nanomedicines combination to treat osteoarthritis. Eur. Polym. J..

[33] Liu X., Wang Z., Qian H., Tao W., Zhang Y., Hu C., Mao W., Guo Q. (2022). Natural medicines of targeted rheumatoid arthritis and its action mechanism. Front. Immunol..

[34] Huria N., Saraf A.A., Padinjarekutt D.L., Gaikwad L., Mourya N., Deo D., Tanpathak S.V., Burande S. (2024). The Biomarker Flavonoid “Rutin” in Morus Species: Isolation, Identification, and Characterization.

[35] Lee Y.J., Jeune K.H. (2013). The effect of rutin on antioxidant and anti-inflammation in streptozotocin-induced diabetic rats. Appl. Microsc..

[36] Meng X.-L., Yu M.-M., Liu Y.-C., Gao Y.-L., Chen X.-S., Shou S.-T., Chai Y.-F. (2022). Rutin inhibits cardiac apoptosis and prevents sepsis-induced cardiomyopathy. Front. Physiol..

[37] Kumar S., Pandey A.K. (2013). Chemistry and biological activities of flavonoids: An overview. Sci. World J..

[38] Kim H.-R., Lee S.-H., Kim Y.-S., Kim S.-N., Kim S.-Y., Park M.H. (2024). *Angelica decursiva* (Miq.) Franch. & Sav. extract inhibits UVB-mediated photoaging by regulating MAPK-related inflammatory pathways. Mol. Cell. Toxicol..

[39] Kim K.S., Choi H.M., Lee Y.-A., Choi I.A., Lee S.-H., Hong S.-J., Yang H.-I., Yoo M.C. (2011). Expression levels and association of gelatinases MMP-2 and MMP-9 and collagenases MMP-1 and MMP-13 with VEGF in synovial fluid of patients with arthritis. Rheumatol. Int..

[40] Xue M., McKelvey K., Shen K., Minhas N., March L., Park S.-Y., Jackson C.J. (2014). Endogenous MMP-9 and not MMP-2 promotes rheumatoid synovial fibroblast survival, inflammation and cartilage degradation. Rheumatology.

[41] García S., Forteza J., López-Otin C., Gómez-Reino J.J., González A., Conde C. (2010). Matrix metalloproteinase-8 deficiency increases joint inflammation and bone erosion in the K/BxN serum-transfer arthritis model. Arthritis Res. Ther..

[42] Harigai M., Kaneko Y., Tanaka E., Hirata S., Kameda H., Kaneko K., Kishimoto M., Kohno M., Kojima M., Kojima T. (2025). 2024 Update of the Japan College of Rheumatology Clinical Practice Guidelines for the Management of Rheumatoid Arthritis-secondary publication. Mod. Rheumatol..

[43] Damerau A., Gaber T. (2020). Modeling rheumatoid arthritis in vitro: From experimental feasibility to physiological proximity. Int. J. Mol. Sci..

[44] Veale D.J., Orr C., Fearon U. (2017). Cellular and molecular perspectives in rheumatoid arthritis. Semin. Immunopathol..

[45] Bartok B., Firestein G.S. (2010). Fibroblast-like synoviocytes: Key effector cells in rheumatoid arthritis. Immunol. Rev..

[46] Bondeson J., Wainwright S.D., Lauder S., Amos N., Hughes C.E. (2006). The role of synovial macrophages and macrophage-produced cytokines in driving aggrecanases, matrix metalloproteinases, and other destructive and inflammatory responses in osteoarthritis. Arthritis Res. Ther..

[47] Pal R.R., Rajpal V., Singh N., Singh S., Mishra N., Singh P., Maurya P., Alka, Saraf S.A. (2023). Downregulation of pro-inflammatory markers IL-6 and TNF-α in rheumatoid arthritis using nano-lipidic carriers of a quinone-based phenolic: An in vitro and in vivo study. Drug Deliv. Transl. Res..

[48] Deng Y., Zheng H., Li B., Huang F., Qiu Y., Yang Y., Sheng W., Peng C., Tian X., Wang W. (2024). Nanomedicines targeting activated immune cells and effector cells for rheumatoid arthritis treatment. J. Control. Release.

