# Supplementary material for: The Therapeutic Effects of Dendropanax morbiferus Lév. Water Leaf Extracts in a Rheumatoid Arthritis Animal Model
Source: Antioxidants (Basel). 2025 May 1;14(5):548. doi: 10.3390/antiox14050548 (PMC12108300; doi:10.3390/antiox14050548)
Supplement: Supplementary file 1 [file antioxidants-14-00548-s001.zip › antioxidants-3570035-supplementary.pptx]

## Slide 1
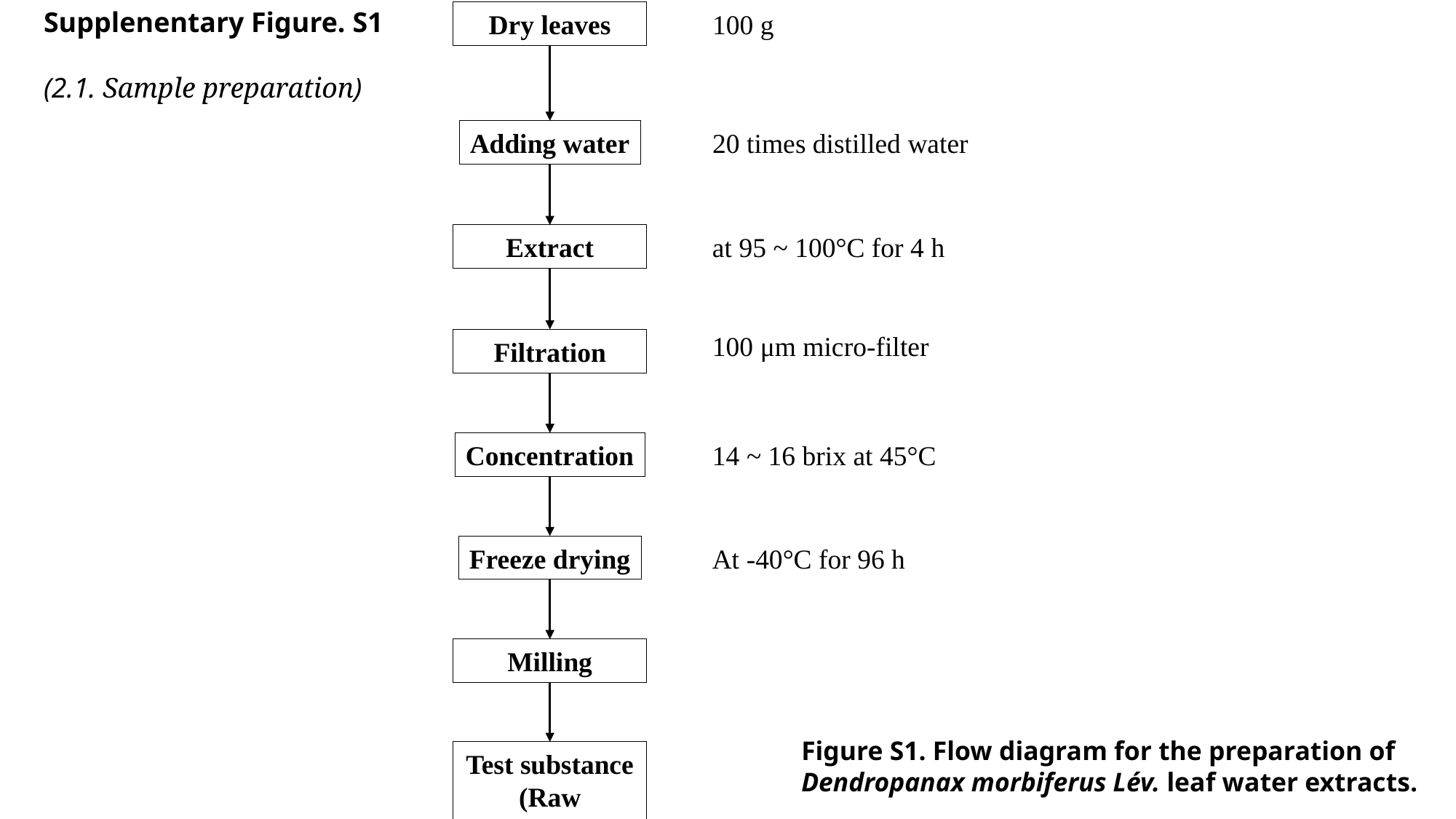

Supplenentary Figure. S1
(2.1. Sample preparation)
Dry leaves
100 g
Adding water
20 times distilled water
Extract
at 95 ~ 100°C for 4 h
100 μm micro-filter
Filtration
Concentration
14 ~ 16 brix at 45°C
Freeze drying
At -40°C for 96 h
Milling
Figure S1. Flow diagram for the preparation of Dendropanax morbiferus Lév. leaf water extracts.
Test substance
(Raw material)

## Slide 2
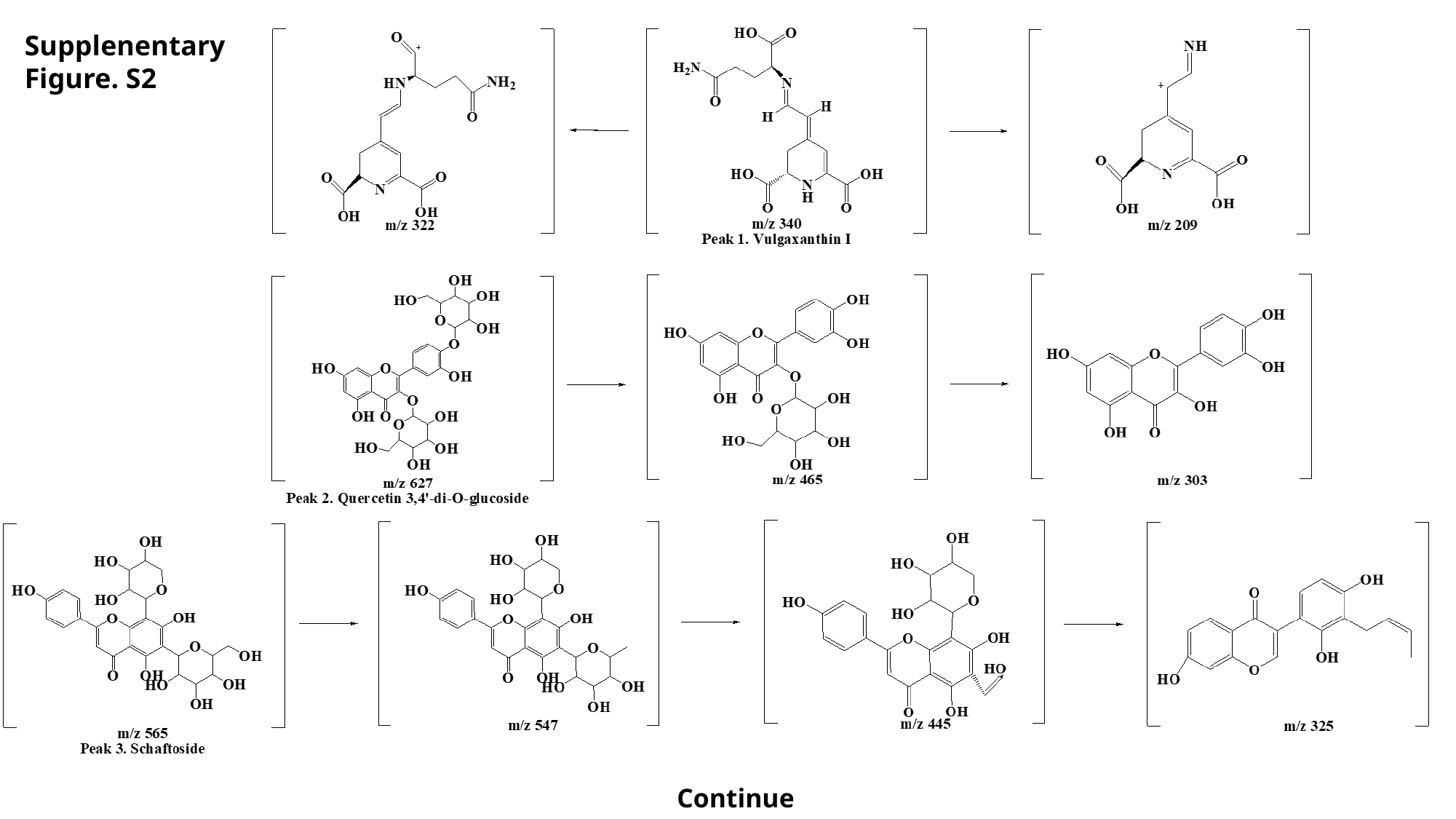

Supplenentary
Figure. S2
Continue

## Slide 3
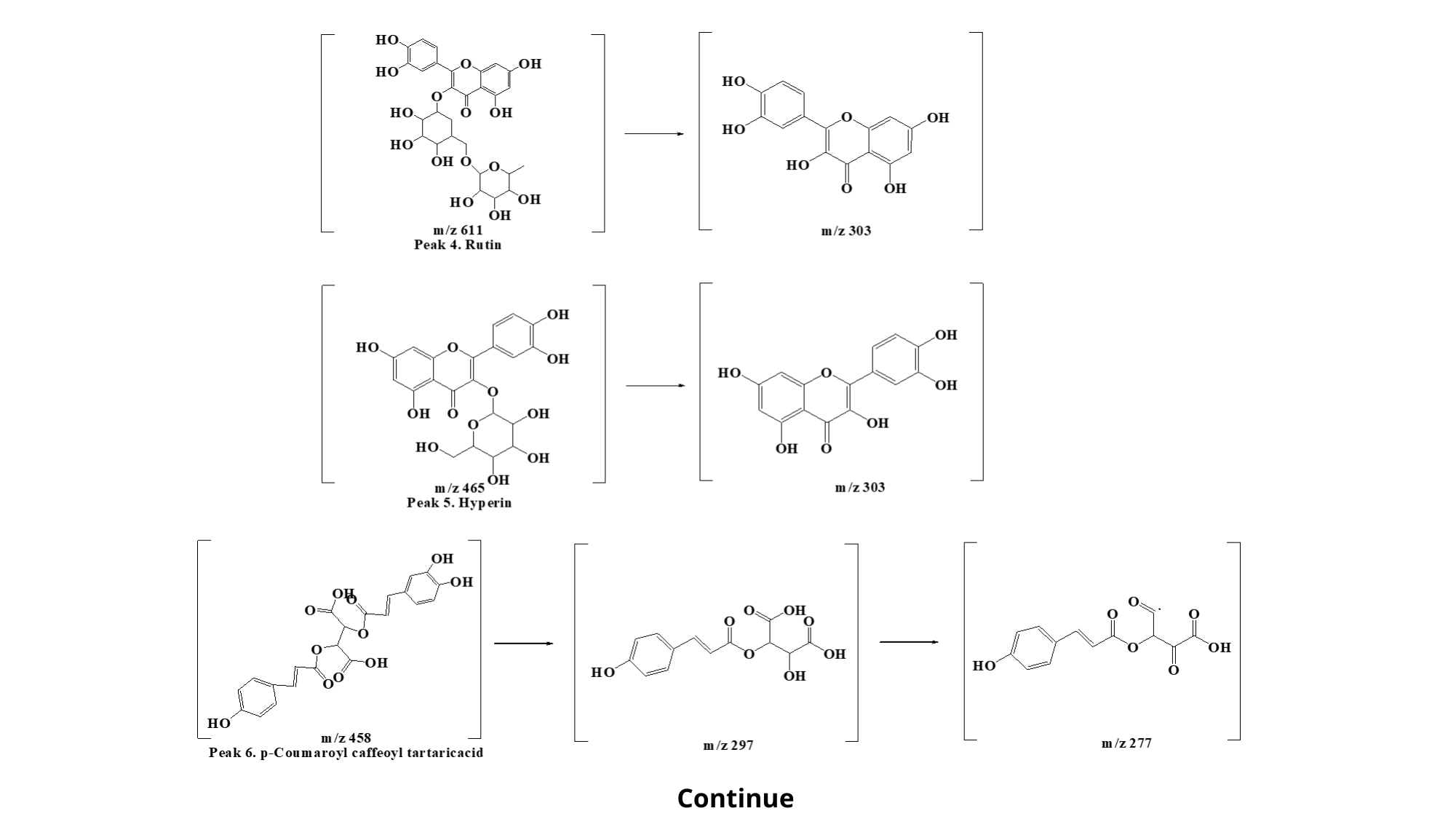

Continue

## Slide 4
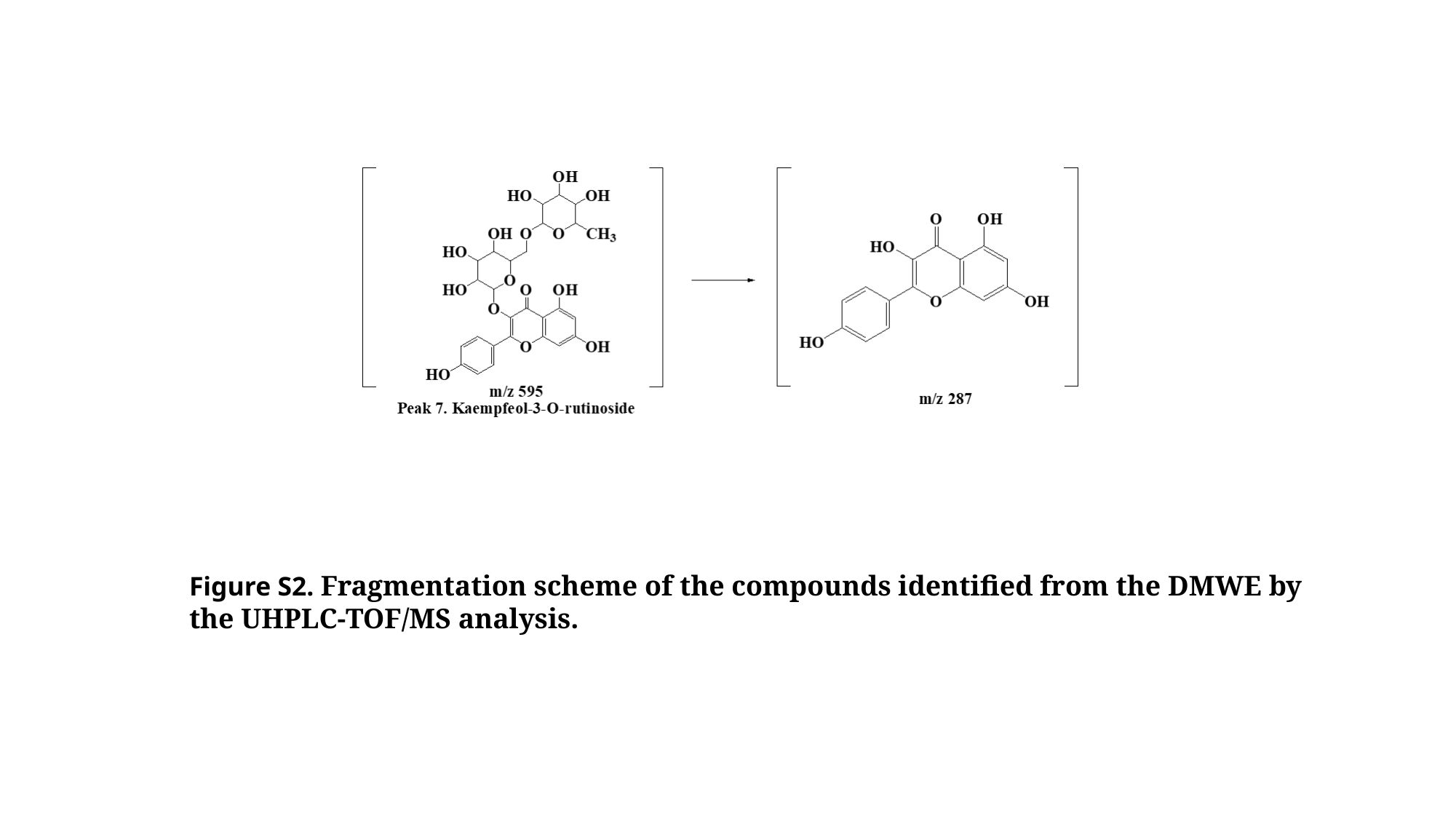

Figure S2. Fragmentation scheme of the compounds identified from the DMWE by the UHPLC-TOF/MS analysis.
